# Case report: An infant boy with X-linked sideroblastic anaemia successfully treated by umbilical cord blood haematopoietic stem cell transplantation

**DOI:** 10.3389/fgene.2022.1009988

**Published:** 2022-11-15

**Authors:** Zhongyang Ma, Dongjun Li, Xue Yang, Juan Liang, Yiping Zhu

**Affiliations:** ^1^ Department of Pediatrics, West China Second University Hospital, Sichuan University, Chengdu, China; ^2^ Key Laboratory of Birth Defects and Related Diseases of Women and Children, Ministry of Education, Sichuan University, Chengdu, China

**Keywords:** congenital sideroblastic anaemia, X-linked sideroblastic anaemia, XLSA, ALAS2, cord blood transplantation, haematopoietic stem cell transplantation, vein occlusion liver disease, case report

## Abstract

X-linked sideroblastic anaemia (XLSA) is an inherited disorder caused by mutations in genes encoding proteins involved in the biosynthesis of haem. The pathogenic gene, as well as the pathogenesis and diagnosis of XLSA, have been fully elucidated in previous studies. However, only a few new advances have been made in managing XLSA in recent years, and blood transfusion remains the primary treatment. We report a case of umbilical cord blood haematopoietic stem cell transplantation in a male infant diagnosed with XLSA who was born with asphyxia due to severe anaemia. Early hepatic vein occlusion occurred after transplantation. However, this complication was rapidly controlled after active treatment, and the child’s quality of life improved significantly. Haematopoietic stem cell transplantation is a promising alternative treatment for XLSA.

## Introduction

XLSA is a most common type of congenital sideroblastic anaemia (CSAs), and is characterised by ring sideroblasts in bone marrow smears stained with Prussian blue (Harigae and Furuyama, 2010; Bottomley and Fleming, 2014). The severity of anaemia varies in XLSA among women and men. Most affected men present with microcytic hypochromic anaemia in the first 2 decades of life, while the women with XLSA more often occur in adulthood and with macrocytic anaemia (Camaschella, 2009; Katsurada et al., 2016; Ducamp and Fleming, 2019). Approximately two-thirds of XLSA cases initially respond to treatment with vitamin B6 but may become unresponsive later, which perhaps due to the exception of the presence of uncontrolled iron overload (Cotter et al., 1999; Astner et al., 2005; Bottomley and Fleming, 2014; Fujiwara and Harigae, 2019). And blood transfusion remains the primary treatment for severe transfusion-dependent XLSA. Herein, we report a case of neonatal asphyxia due to severe anaemia, later confirmed by genetic examination as XLSA. We treated the patient using haematopoietic stem cell transplantation (HSCT) when he was 1 year old. And this is the first case of XLSA treated with HSCT.

## Case presentation

The patient was 13 months old and was admitted to our hospital for skin pallor since birth. He was born *via* emergency caesarean section due to foetal distress. There were no obstetric complications, including placental abruption or bloody amniotic fluid. He was born with pale skin, perioral cyanosis, poor reaction, and dyspnoea. His Apgar scores at one, five, and 10 minutes were 5, 7, and 9, respectively. After resuscitation in the delivery room, he was transferred to the neonatology department of the local hospital. A routine blood examination showed a haemoglobin concentration of 9.5 g/dl. His condition was diagnosed as neonatal anaemia and neonatal asphyxia, and he was placed on a non-invasive ventilator. He received a red blood cell transfusion, which increased his haemoglobin concentration to 12.5 g/dl. There is no consanguinity in his parents, and neither had a history of anaemia. He was discharged at 14 days of age. One month later, his skin pallor resumed, accompanied by hyporeactivity and reduced appetite, without the presence of yellow skin, haematochezia, or haematemesis. He was presented to his doctor and was diagnosed with severe anaemia, as his haemoglobin concentration was only 5.0 g/dl. Gene sequencing identified a mutation in the ALAS2 gene, leading to the diagnosis of X-linked sideroblastic anaemia when he was 3 months old (Table 1). He received a blood transfusion of approximately 15 ml of red blood cells per kilogram of body weight. Thereafter, his skin became rubicund, and he became more energetic. Regular blood transfusions at 2–4 weeks intervals were subsequently performed in the local hospital at 15 ml red blood cells per kilogram of body weight. The haemoglobin concentration was 7.0–8.0 g/dl before transfusion and 10.0–12.0 g/dl after transfusion. The patient was maintained on regular blood transfusions. However, he had feeding difficulties (difficulty consuming complementary food) 6 months before admission (at 7 months of age) and showed retarded growth and development. He weighed 7.2 kg (the 10th percentile of weight for his age and sex). His height was 66 cm (approximately the 10th–25th percentile of height for his age and sex). He was referred to our hospital for further evaluation.

**TABLE 1:** Details of the laboratory examination before hematopoietic stem cell transplantation and results of gene sequencing.

Item	Value	Reference values(ranges)
**Complete blood count**		
WBC	6.6×10^9^/L	4.4-11.9×10^9^/L
PLT	411×10^9^/L	125-462×10^9^/L
RBC	4.09×10^12^/L	4.0-5.5×10^12^/L
MCV	71.4fL	80-10fL
MCH	21.0pg	28-32pg
MCHC	295g/L	320-360g/L
HGB	86g/L	110-150g/L
Reticulocyte count	0.8×10^9^/L	24-84×10^9^/L
Reticulocyte%	0.02%	0.5-1.5%
**Bio-chemistry**		
ALT	24U/L	<49U/L
AST	27U/L	<40U/L
Total bilirubin	4.9μmol/L	5-23μmol/L
Conjugated Bilirubin	2.2μmol/L	<8μmol/L
**Indexes of iron metabolism**		
Ferritin	551.4ng/ml	22-322ng/ml
Serum iron	25.9μmol/L	10.6-36.7μmol/L
Transferrin	1.59g/L	2.0-3.6g/L
Transferrin saturation	74.9%	20-55%
Total iron binding capacity	34.6μmol/L	44.6-69.3μmol/L
**Blood group Antibody screening**		
Direct antiglobulin test	Negative	
Indirect antiglobulin test	Negative	
Isopropanol test	Negative	
**hemoglobin electrophoresis**		
HbF	1.2%	
HbA_2_	2.7%	
G-6-PD	6.6u/gHG	
**Details of the gene sequencing in the patient’s family**		
Gene	ALAS2	
Location of chromosomal	chrX:55047615	
Transcript number	NM_000032; exon5	
Change of nucleotide	c.508C>T	
Change of Amino acid	p.R170C	
Types of gene mutation	missense mutation	
Mode of inheritance	X-linked	
**The mother’s genotype as heterozygous for c.508C>T, p.R170C**		

Abbreviations: WBC, white blood cell, PLT, blood platelet, RBC, red blood cell, MCV: merkel cell polyomavirus, MCH, Melanin-concentrating hormone, MCHC, mean corpuscular hemoglobin concentration, HGB, hemoglobin, ALT, alternative lengthening of telomeres; AST, aspartate aminotransferase.

## Diagnostic assessment

Blood tests and bone marrow smears were performed in the outpatient department of our hospital. Detailed examination reports are shown in Table 1.

The bone marrow smear showed ring sideroblasts. Blood tests for ferritin, the Coombs test, and haemoglobin electrophoresis ruled out iron deficiency anaemia, major thalassaemic anaemia, and other common microcytic hypochromic anaemia disorders. These results, combined with the gene sequencing report, led to the diagnosis of XLSA. The iron metabolism index was monitored during the patient’s regular blood transfusions in the outpatient department. The latest ferritin concentration before HSCT was 562.9 ng/ml, and the transferrin saturation was 74.9%. The feeding difficulties caused by chronic anaemia, retarded growth and development, the high cost of long-term blood transfusions, and the high risks associated with blood transfusions seriously affected the lives of his family. The parents strongly requested HSCT. However, the patient had no potential sibling donors, and no matching unrelated homozygous allogeneic healthy donors were identified, when unrelated HLA 10/10 identified umbilical cord blood stem cells were searched.

At admission, the patient was 13 months old, weighing 9.3 kg (slightly higher than P15), with a height of 72 cm (less than P3). He had mild anaemic symptoms and nail pallor but lacked notable facies and yellow skin or sclera. No skin rash, subcutaneous haemorrhagic spots, or petechiae were observed. There was no palpable enlargement of superficial lymph nodes. The head circumference was 45 cm (close to P50), the anterior fontanelle was approximately 0.5 × 0.5 cm, and the tension was not high. The entire abdomen was soft, with an abdominal circumference of 40 cm, and no palpable mass was observed. The liver was palpable 2.5 cm under the ribs, with soft and sharp edges, while the spleen was not palpable. Examination findings for the cardiopulmonary and nervous systems were normal. No limb deformity was observed. All pretransplant examinations performed after admission were normal, including abdominal ultrasound, abdominal computed tomography, and liver function.

## Therapeutic intervention and outcomes

A myeloablative conditioning regimen was administered (busulfan, 1.2 mg/kg for 3 days; fludarabine, 30 mg/m^2^ for 4 days; thiotepa, 5 mg/kg for 2 days; and cyclophosphamide, 60 mg/kg for 2 days). Transfusion of 25 ml of unrelated HLA 10/10 fully matched cord blood stem cells was performed (CD34^+^ cell count, 4.23×10^5^/kg; TNC, 10.12×10^7^/kg; MNC, 3.72×10^7^/kg). After transplantation, mycophenolate mofetil and tacrolimus were administered to prevent graft-versus-host disease (GVHD). Granulocyte colony-stimulating factor and recombinant thrombopoietin were used to promote haematopoietic reconstitution. Prostaglandin E1 was applied to prevent liver veno-occlusive disease (VOD).

**FIGURE 1:**
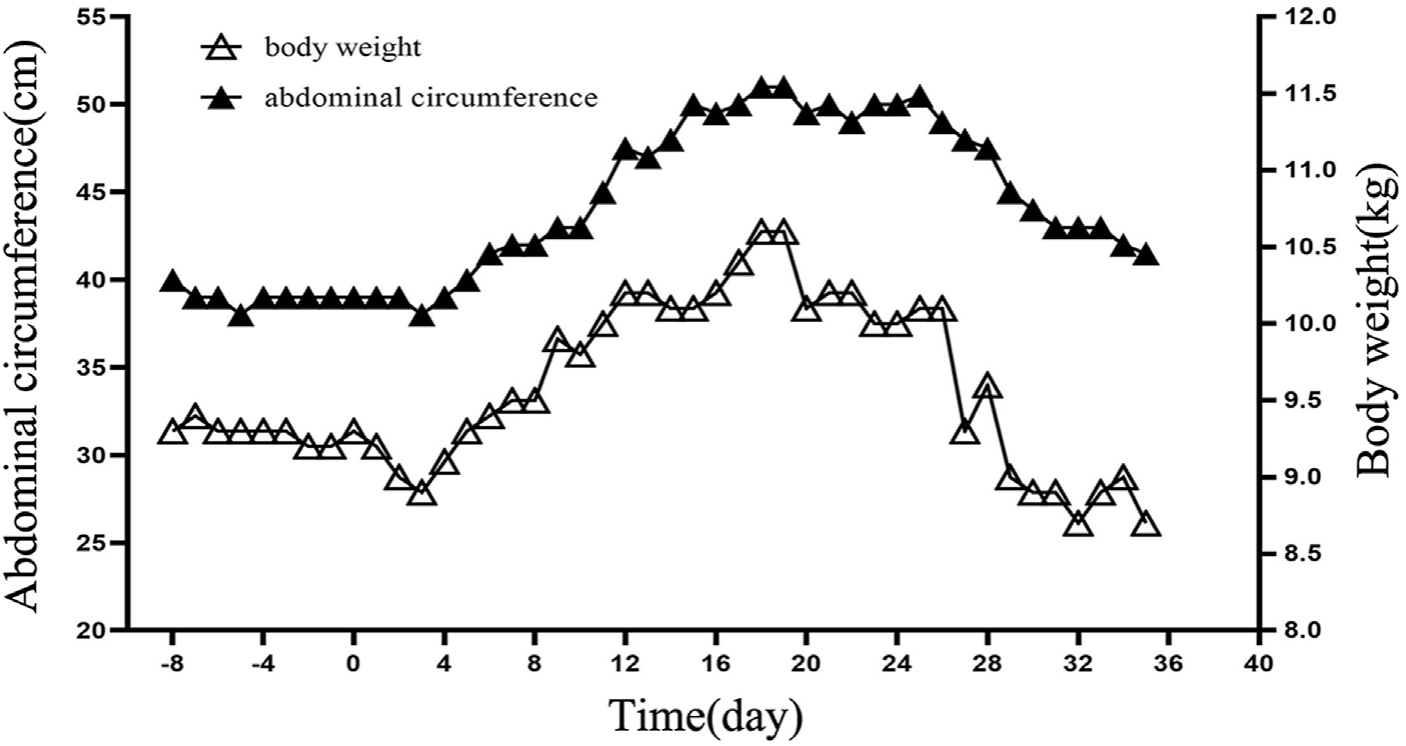
Details of changes in body weight and abdominal circumference. The *X*-axis represents time, with negative values indicating days before transplantation and positive values indicating days after transplantation. For example, −8 for 8 days before transplantation and 20 for 20 days after transplantation. The *Y*-axis on the left represents the abdominal circumference in centimetres. The Y-axis on the right represents the weight in kilograms.

**FIGURE 2:**
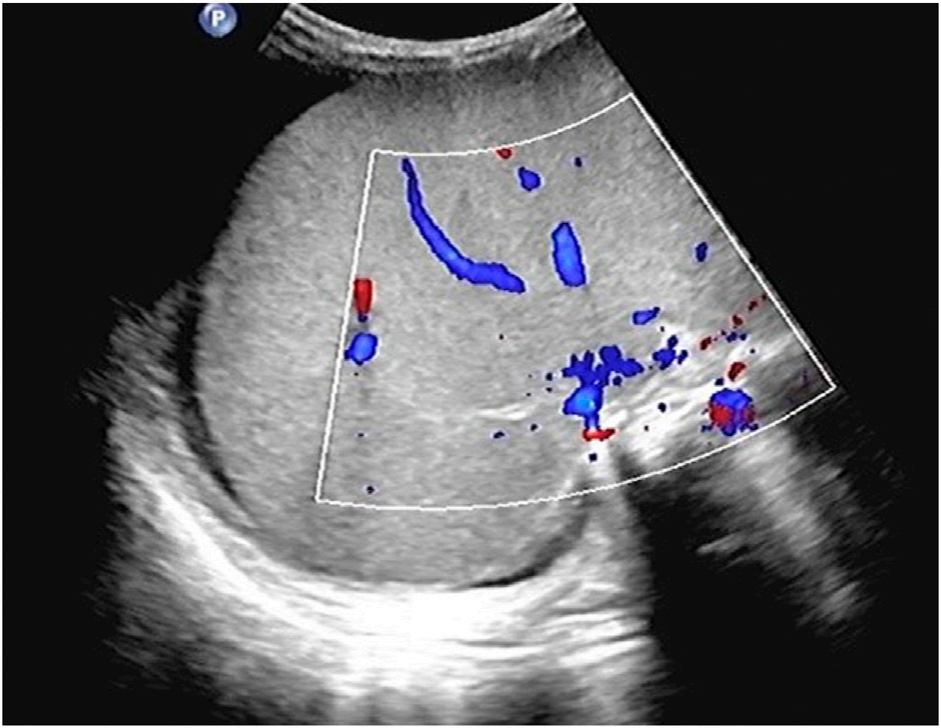
Results of abdominal ultrasound examination suggest ascites and right hepatic vein occlusion. There was an anechoic area around the liver, and the right hepatic vein lumen was not clearly displayed, and there was no obvious blood flow signal in the right hepatic vein course area.

The patient showed a progressive increase in body weight and abdominal circumference on day 11 after transplantation. By day 19 post-transplantation, both body weight and abdominal circumference reached their maximum increase, with a 14.0% increase in body weight and a 27.5% increase in abdominal circumference compared with the data on admission (Figure 1). Physical examination showed that the liver was progressively enlarged. The liver was palpable 7 cm below the ribs, with a slightly tough texture, round blunt edge, and a smooth surface. A liver function examination revealed that the direct bilirubin level was 34.3 μmol/L, and the albumin level was 29.8 g/L. Ultrasound suggested an enlarged liver, ascites, and right hepatic vein occlusion (Figure 2). At the time, granulocytes and platelets had not been engrafted, and there were no obvious infections, acute GVHD, or other manifestations. Refractoriness to platelet transfusion was a novel manifestation. Combined with the above symptoms, we considered the presence of VOD. Fluid restriction, diuresis, and an increase in the dose of prostaglandin E1 were used as first-line treatment; however, there was no improvement in the patient’s condition. Defibrotide(25 mg/kg/d, divided into four doses) was used on day 23 post-transplantation. After 4 days of defibrotide treatment, urine volume increased significantly, and the body weight and abdominal circumference began to decrease. The body weight decreased to the pretransplantation level on day 34 after transplantation, and the abdominal circumference was stable at approximately 44 cm. Repeat ultrasound suggested that the ascites had been absorbed.

Neutrophils were engrafted 25 days after transplantation, and platelets were engrafted 48 days after transplantation. The patient’s peripheral blood chimerism at 56 days post-transplantation showed 100% of whole blood and 100% of T-cells. Currently, 5 months post-transplantation, the patient is regularly followed up at our outpatient clinic and has shown no occurrence of HSCT-associated complications. The patient can also consume rice noodles, vegetables, and fruit juice as supplementary foods, and his weight has increased over time.

## Discussion

Genetic testing is becoming increasingly important in analysing the aetiology of severe anaemia at birth, especially in neonates, with the exclusion of some common causes of anaemia, such as blood loss due to an acute event at birth and insufficient erythropoiesis synthesis due to maternal malnutrition during pregnancy. The presence of anaemia caused by congenital genetic diseases, such as thalassemia, pyruvate kinase deficiency, and XLSA, should be highly suspected in these patients (Ma and Yang, 2021; Makis et al., 2021; Tangsricharoen et al., 2021). Analysing the aetiology of these diseases often requires functional tests, such as metabolic enzyme activity or haemoglobin electrophoresis. However, severe anaemia at birth is a critical condition that usually requires resuscitation and even blood transfusion when it is urgently required to maintain stable vital signs, which may lead to false-negative test results. Additionally, newborns have special physiological states, which may lead to interference, and functional tests require many blood samples. These factors make it difficult to inspect enzyme activity in red blood cells and conduct haemoglobin electrophoresis immediately (Kalteren et al., 2018). Thus, traditional testing methods are of limited value for the aetiological analysis of severe anaemia at birth.

However, gene sequencing only requires approximately 1–2 ml of blood, and the result is not affected by blood transfusion and other treatments, which may facilitate early diagnosis of the disease. We previously reported a case of severe anaemia at birth, initially diagnosed as congenital pyruvate kinase deficiency based on gene sequencing, which was cured by unrelated, fully matched peripheral blood stem cell transplantation (Ma and Yang, 2021). The patient in this case also had severe anaemia at birth, accompanied by apnoea which required non-invasive ventilator-assisted ventilation. Gene sequencing revealed a missense mutation in the ALAS2 gene located on the X chromosome, thus confirming the diagnosis of XLSA. ALAS2 catalyses the first and rate-limiting steps in haem synthesis (condensation of glycine and succinyl CoA to δ-aminolaevulinic acid), which is critical for erythroid cell development. Its mutation results in a red blood cell maturation disorder manifested as XLSA (Kaneko et al., 2014; Fujiwara and Harigae, 2019).

Blood transfusion is currently the primary treatment for children with sever transfusion-dependent XLSA who do not respond to vitamin B6. However, long-term transfusions carry a risk of transfusion-related complications and iron overload, which can lead to organ dysfunction. The patient, in this case, showed retarded growth and development caused by chronic anaemia, and the long-term treatment disturbed the lives of his family and resulted in an economic burden. Therefore, his parents urgently chose HSCT to cure his condition. HSCT is a radical treatment for various inherited anaemias, such as major thalassemia; Some previous studies have reported that HSCT successfully cured transfusion-dependent CSA, but none of the patients in these studies had genetic testing (Urban et al., 1992; Ayas et al., 2001; Meo et al., 2006). Heeney et al. reported nine CSA with mutaion of SLC25A38 gene have undergone HSCT. All transplanted patients are alive, eight of nine had became transfusion independent, while one patient had secondary graft failure at 18 months posttransplant (Heeney et al., 2021). And Uminski K et al. reported a significant iron loading CSA patient with mutaion of SLC25A38 gene died in the posttransplant period due to complications of infections. So they made a conclusion that timely HSCT can result in excellent long-term outcomes in CSA (Uminski et al., 2020). However, there is no report about HSCT in XLSA to date. We selected umbilical cord blood stem cells for this patient for the following reasons: 1) he had no siblings, and we failed to find a matched unrelated peripheral blood stem cell donor; and 2) he was young and had relatively low weight and the number of stem cells in the umbilical cord blood met the transplant standards. Fortunately, unrelated, fully matched umbilical cord blood stem cells that met the transplant standards were available. Additionally, the patient’s parents agreed to the transplant willingly and fully understood the risks and complications of transplantation. Current studies show that the failure of umbilical cord blood stem cell engraftment is mainly related to the number of stem cells (Li et al., 2018; Parikh et al., 2021). In this case, neutrophils and platelets were engrafted 25 and 48 days after transplantation, respectively. The patient’s peripheral blood chimerism at 56 days after transplantation showed 100% of whole blood and 100% of T-cells, all of which suggested successful engraftment.

Unfortunately, VOD was diagnosed after transplantation, and conventional and cost-effective treatments were ineffective. Defibrotide treatment was administered, which rapidly controlled VOD and stabilised haematopoietic reconstitution. A review of the entire course of the disease, including follow-up, showed that VOD did not affect the patient’s quality of life, and his later regular liver function assessments were normal. VOD is a severe complication of HSCT. Iron overload before transplantation, abnormal liver function, and high-dose chemotherapy during preconditioning are the risk factors for VOD (Dalle and Giralt, 2016; Bonifazi et al., 2020). In this case, liver function examination and liver imaging findings of the patient before transplantation were normal, and the chemotherapy regimen was standard. Therefore, repeated blood transfusions after birth and excess tissue iron deposition caused by his condition may have contributed to VOD. We concluded that the patient, in this case, benefited from HSCT, possibly because of early transplantation. If the timing of transplantation was delayed to when he was 2 years old or later, VOD could have worsened and become more challenging to treat due to organ dysfunction caused by anaemia and more severe iron overload. This could have resulted in persistent organ dysfunction and even death.

In conclusion, HSCT could be used to treat transfusion-dependent XLSA, and the time of transplantation should be earlier rather than later. Well-matched, unrelated cord blood stem cells could be considered for patients without a sibling donor. But more researches are needed to verify this conclusion.

## Patient perspective

The patient was too young to express his view on HSCT. However, before transplantation, we fully communicated the potential benefits and risks of regular blood transfusion and HSCT to his parents. They understood the condition and signed a written consent form accepting HSCT. Although VOD occurred after transplantation, they are very satisfied with the overall outcome, believing that HSCT has improved the patient’s condition and quality of life.

## Data Availability

The original contributions presented in the study are included in the article/supplementary material, further inquiries can be directed to the corresponding author.
